# Steroid exposure and outcome in COVID-19 pneumonia

**DOI:** 10.1016/j.bjao.2023.100128

**Published:** 2023-01-31

**Authors:** Christopher Remmington, Nicholas A Barrett, Sangita Agarwal, Boris Lams, Patrick Collins, Valentina Camarda, Chris Meadows, Fraser Hanks, Barnaby Sanderson, Andrew Retter, Luigi Camporota

**Affiliations:** 1 Pharmacy Department, Guy's and St Thomas' NHS Foundation Trust, London, UK; 2 Institute of Pharmaceutical Science, King's College London, London, UK; 3 Department of Adult Critical Care, Guy's and St Thomas' NHS Foundation Trust, London, UK; 4 Centre for Human & Applied Physiological Sciences (CHAPS), School of Basic & Medical Biosciences, Faculty of Life Sciences & Medicine, King's College London, London, UK; 5 Department of Rheumatology and Guy's and St Thomas' NHS Foundation Trust, London, UK; 6 Department of Respiratory Medicine, Guy's and St Thomas' NHS Foundation Trust, London, UK

**Keywords:** acute respiratory distress syndrome, corticosteroid, COVID-19, mechanical ventilation, steroid

## Abstract

**Background:**

Corticosteroids are used to treat COVID-19 pneumonia. However, the optimal dose is unclear. This study describes the association between corticosteroid exposure with disease severity and outcome in COVID-19 pneumonia.

**Methods:**

This is a single-centre retrospective, observational study including adult ICU patients who received systemic corticosteroids for COVID-19 pneumonia between March 2020 and March 2021. We recorded patient characteristics, disease severity, total steroid exposure, respiratory support and gas exchange data, and 90-day mortality.

**Results:**

We included 362 patients. We allocated patients to groups with increasing disease severity according to the highest level of respiratory support that they received: high-flow nasal oxygen or continuous positive airway pressure (HFNO/CPAP) in 12.7%, invasive mechanical ventilation (IMV) in 61.6%, and extracorporeal membrane oxygenation (ECMO) in 25.7%. For these three groups, the median (inter-quartile range [IQR]) age was 61 (54–71) *vs* 58 (50–66) *vs* 46 (38–53) yr, respectively (*P*<0.001); median (IQR) APACHE (Acute Physiology and Chronic Health Evaluation) II scores were 12 (9–15) *vs* 14 (12–18) *vs* 15 (12–17), respectively (*P*=0.006); the median (IQR) lowest $Pa_{O_{2}}$/FiO_2_ ratio was 15.1 (11.8–21.7) *vs* 15.1 (10.7–22.2) *vs* 9.5 (7.9–10.9) kPa, respectively (*P*<0.001). Ninety-day mortality was 9% *vs* 27% *vs* 37% (*P*=0.002). Median (IQR) dexamethasone-equivalent exposure was 37 (24–62) *vs* 174 (86–504) *vs* 535 (257–1213) mg (*P*<0.001). ‘Pulsed’ steroids were administered to 26% of the IMV group and 48% of the ECMO group. Patients with higher disease severity who received pulse steroids had a higher 90-day mortality.

**Conclusions:**

Corticosteroid exposure increased with the severity of COVID-19 pneumonia. Pulsed dose steroids were used more frequently in patients receiving greater respiratory support. Future studies should address patient selection and outcomes associated with pulsed dose steroids in patients with severe and deteriorating COVID-19 pneumonia.

The pathogenesis of respiratory failure caused by coronavirus–19 disease (COVID-19) is thought to involve direct viral injury and consequent activation of a dysregulated systemic and pulmonary inflammatory response leading to endothelial injury, hypercoagulability and thrombosis.^1^^,^^2^ In more severe COVID-19, pulmonary injury progresses to acute respiratory distress syndrome (ARDS), characterised by interstitial and alveolar infiltrates, hypoxaemia, and diffuse alveolar damage.^3^

**Figure 1:**
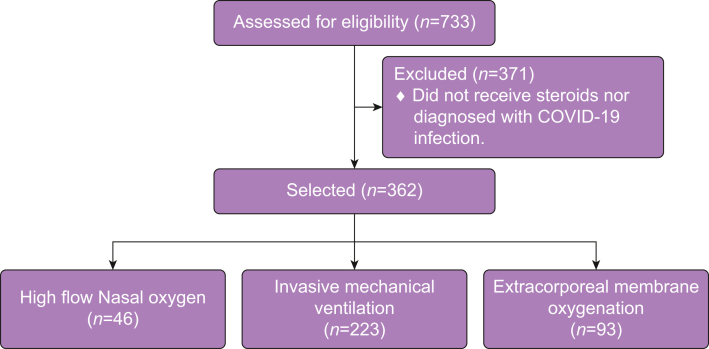
Steroid exposure and outcome in patients with COVID-19 pneumonia.

Corticosteroids are recommended in hospitalised COVID-19 patients receiving oxygen therapy or higher-level respiratory support.^4^, ^5^, ^6^ The Randomised Evaluation of Covid-19 Therapy (RECOVERY) trial demonstrated that dexamethasone 6 mg daily for 10 days reduced 28-day mortality and hospital length of stay, and the beneficial effect of dexamethasone was greater in patients receiving invasive mechanical ventilation (IMV).^4^

The relationship between the severity of respiratory failure, the dose of steroids used, and outcome in COVID-19 is still not well defined. Patients in the RECOVERY trial received a significantly lower dose of dexamethasone than those with ARDS enrolled in other trials.^7^, ^8^, ^9^ In particular, in the DEXA-ARDS trial, in patients with non-COVID ARDS ($Pa_{O_{2}}$/FiO_2_ [PF], ≤26.6 kPa) investigators demonstrated an increase in ventilator-free days and reduced 60-day mortality in patients receiving dexamethasone 20 mg daily for 5 days, followed by 10 mg daily for 5 days.^9^ Furthermore, for patients with COVID-19 ARDS (C-ARDS), the 7–10-day course of steroids used in these trials is shorter than that previously advised in ARDS (up to 28 days).^10^ Other trials have explored higher doses for late unresolved disease (>7 days), in non-COVID ARDS (2 *vs* 1 mg kg^−1^ methylprednisolone).^10^ The higher doses of steroids may be justified by the fact that C-ARDS may have pathological and radiological features of organising pneumonia,^11^^,^^12^ where steroids, including ‘pulsed dose’ steroids (methylprednisolone >250 mg daily) have been considered the standard of care.^13^ There has also been a small RCT of pulsed methylprednisolone in patients which demonstrated a mortality benefit in patients with C-ARDS who required oxygen therapy but not IMV or veno-venous extracorporeal membrane oxygenation (V-V ECMO).^14^

The primary objective of this study was to describe the association between the cumulative corticosteroid exposure in patients with C-ARDS with disease severity and outcome. The secondary objective was to describe the population risk factors for receiving pulsed methylprednisolone and 90-day mortality.

## Methods

### Study design and participants

The study was a retrospective, observational review of prospectively identified patients. Recruitment took place from 1 March 2020 to 31 March 2021 on the critical care units at Guy's and St Thomas' NHS Foundation Trust a tertiary university affiliated hospital with a regional extracorporeal membrane oxygenation (ECMO) retrieval service. Participants were included if aged 18 years and older and they received corticosteroids (dexamethasone, hydrocortisone, methylprednisolone, and prednisolone) for C-ARDS. Patients were categorised by the highest level of respiratory support they received during admission, high-flow nasal oxygen or continuous positive airway pressure (HFNO/CPAP), IMV, or ECMO.

### Study approvals and data collection

Institutional approval was gained from the local quality improvement and safety committee (reference number: 12867). Informed consent was waived for this retrospective analysis of data collected prospectively for routine care, without breach of privacy or anonymity. The study qualified as a service evaluation as defined by the UK NHS Health Research Authority and therefore did not require review by a research ethics committee.

Data were extracted from the electronic health record system (Philips ICIP-IntelliSpace CCA) and ancillary service provision databases of two hospitals at Guy's and St Thomas' NHS Foundation Trust.

### Variables

The primary outcome was defined as 90-day mortality and disease severity was defined by the highest level of respiratory support on admission to the tertiary centre. Steroid dose administration was tallied during critical care admission in this institution only – steroid doses given before admission for patients transferred from another institution were not available. Corticosteroid exposure, defined as dexamethasone dose equivalent and expressed in milligrams (mg), was calculated in accordance with previous studies.^15^, ^16^, ^17^ ‘Pulsed’ intravenous methylprednisolone was defined as a dose of methylprednisolone >250 mg daily for 3 days. Dexamethasone exposure per day was calculated by tallying the total steroid dose divided by the number of days receiving steroid therapy. Biochemistry and ventilation data were collected within 24 h of admission to the tertiary hospital, except for patients retrieved on ECMO where data were collated from referral data before commencing ECMO support. Physiological and ventilation data were summarised as the worst values recorded for each patient. Missing data were omitted. The length of hospital stay for patients supported with ECMO is limited to the period of time at our institution. The period of admission before transfer or after transfer back to the referring institution was not available.

### Statistical analysis

Categorical data are presented as counts (%), and comparisons were made using Wilcoxon rank sum or Pearson χ^2^ tests. Continuous data are presented as medians and inter-quartile range. The Kruskal–Wallis test was used to compare continuous variables across the three respiratory support groups. Univariable logistic regression was performed on appropriate covariates to examine the association with two outcomes: receiving pulsed i.v. methylprednisolone and 90-day mortality. Multivariable logistic regression was performed for 90-day mortality. All tests were two-tailed, with *P*<0.05 considered statistically significant. Data analysis was performed using R Statistical Software (v4.2; R Core Team 2021, R Foundation for Statistical Computing, Vienna, Austria).

## Results

### Patient cohort: characteristics

We included 362 patients with confirmed COVID-19 pneumonia (Fig. 1). Baseline characteristics of the whole cohort are reported in Table 1. Patients were grouped based on the highest level of respiratory support they received during admission (Table 1). There were 46 patients (12.7%) supported with HFNO or CPAP, 223 (61.6%) patients supported with IMV, and 93 (25.7%) with ECMO.

**Table 1:** Comparison of patient characteristics and disease severity at admission. ∗Continuous data are presented as median (IQR). Categorical data presented as counts/total count (%). ^†^Kruskal–Wallis test to compare difference across groups. ^‡^Steroid dose as dexamethasone equivalence in milligrams. APACHE, Acute Physiology and Chronic Health Evaluation; CPAP; continuous positive airway pressure; ECMO, extracorporeal membrane oxygenation; HFNO, high-flow nasal oxygen; IQR, inter-quartile range; IVMP, intravenous methylprednisolone; NA, not available; PBW, predicted body weight; Pulse, receiving >250 mg daily for 3 days.

Characteristic	HFNO/CPAP∗ (*n*=46)	IMV∗ (*n*=223)	ECMO∗ (*n*=93)	*P*-value^†^
Age (yr)	61 [54, 71]	58 [50, 66]	46 [38, 53]	<0.001
Sex - male, n (%)	34 [73.9]	153 [68.6]	63 [67.7]	0.744
Weight (kg)	78 [70, 95]	84 [75, 100]	90 [80, 108]	0.004
APACHE II	12 [9, 15]	14 [12, 18]	15 [12, 17]	0.006
$Pa_{O_{2}}$/FiO_2_ (kPa)	15.1 [11.8, 21.7]	15.1 [10.7, 22.2]	9.5 [7.9, 10.9]	<0.001
C-reactive protein (mg L^−1^)	70 [36, 154]	133 [70, 224]	186 [80, 319]	<0.001
Procalcitonin (μg L^−1^)	0.17 [0.10, 0.36]	0.59 [0.2, 1.61]	0.9 [0.22, 2.46]	<0.001
Lymphocytes (10×^9^ L^−1^)	0.7 [0.5, 0.8]	0.7 [0.5, 1.0]	0.6 [0.4, 0.90]	0.289
*Respiratory variables*				
FiO_2_ (%)	0.7 [0.6, 0.8]	0.7 [0.6, 0.8]	0.90 [0.80, 1.00]	<0.001
$Pa_{O_{2}}$ (kPa)	8.47 [7.54, 8.90]	8.60 [7.69, 10.04]	8.50 [7.32, 9.55]	0.204
$Pa_{{CO}_{2}}$ (kPa)	4.82 [4.47, 5.30]	6.10 [5.24, 7.35]	7.49 [6.76, 9.00]	<0.001
pH	7.44 [7.41, 7.47]	7.36 [7.30, 7.40]	7.33 [7.26, 7.39]	<0.001
SaO_2_ (%)	91.6 [89.1, 93.6]	91.3 [89.3, 93.7]	85.0 [73.5, 91.0]	<0.001
Lactate (mmol L^−1^)	1.9 [1.4, 2.5]	1.6 [1.2, 2.1]	1.3 [1.0, 1.9]	<0.001
Bicarbonate (mmol L^−1^)	22.9 [20.6, 24.9]	25.4 [21.7, 28.0]	27.0 [23.8, 30.9]	<0.001
Base excess (mmol L^−1^)	–0.9 [–2.8, 0.9]	–0.7 [–3.0, 3.0]	4.5 [1.8, 8.3]	<0.001
HFNO/CPAP flow rate (L min^−1^)	50 [50, 50]	NA	NA	
Ventilatory frequency (bpm)	28 [25, 32]	20 [18, 24]	22 [18, 25]	<0.001
Tidal volume (ml)	684 [662, 705]	542 [474, 630]	460 [400, 509]	<0.001
Tidal volume per PBW (ml kg^−1^)	NA	8.6 [7.3, 9.8]	7.2 [6.0, 8.6]	<0.001
Peak airway pressure (cm H_2_O)	NA	24 [24, 24]	32 [29, 38]	0.176
PEEP (cm H_2_O)	5 [4.5, 6.5]	10 [8, 12]	12 [10, 14]	<0.001
Driving pressure (cm H_2_O)	NA	16.0 [13.5, 20.0]	17.0 [14.0, 21.0]	0.349
Minute volume (L min^−1^)	12.4 [11.3, 13.6]	9.2 [7.7, 10.9]	9.6 [7.7, 10.9]	0.320
Pressure support (cm H_2_O)	16 [16, 16]	12 [8, 16]	NA	0.349
Compliance (ml cm H_2_O^−1^)	NA	32 [23, 47]	29 [20, 35]	0.022
*Outcome variables*				
ICU length of stay (days)	5 [4, 7]	28 [16, 44]	24 [13, 44]	<0.001
90-day mortality	4 [8.7]	61 [27.3]	34 [36.6]	0.002
Discharge from hospital after 90 days	42 [91.3]	160 [71.7]	55 [59.1]	<0.001
*Corticosteroid variables*^‡^				
Total steroid exposure	37 [24, 62]	174 [86, 504]	535 [257, 1213]	<0.001
Steroid duration (days)	6 [4, 8]	18 [10, 28]	20 [11, 30]	<0.001
Average daily steroid dose	7 [6, 8]	11 [7, 19]	25 [17, 39]	<0.001
Total steroids dose without pulse	37 [24, 62]	158 [82, 294]	375 [244, 612]	<0.001
Total steroid days without pulse	6 [4, 8]	17 [10, 28]	19 [11, 28]	<0.001
Average daily steroid dose without pulse	7 [6, 8]	9 [7, 13]	19 [14, 25]	<0.001
Number of patients receiving Pulse methylprednisolone	0 [0]	58 [26.0]	45 [48.4]	<0.001
Time to IVMP pulse from admission (days)	NA	10 [4, 16]	11 [4, 17]	0.956

Patients receiving higher levels of support described in Table 1 (ECMO *vs* IMV *vs* HFNO/CPAP) were younger, with greater body weight, with lower $Pa_{O_{2}}$/FiO_2_ ratio, higher $Pa_{{CO}_{2}}$, higher Acute Physiology and Chronic Health Evaluation (APACHE) II score, higher C-reactive protein (CRP), and higher procalcitonin (PCT).

### Corticosteroid exposure, disease severity, and outcome

Corticosteroid exposure (total exposure and duration of treatment) increased with disease severity (Table 1). The median (inter-quartile range [IQR]) total steroid exposure (including pulsed i.v. methylprednisolone) in the HFNO/CPAP, IMV, and ECMO groups was 37 (26–62) *vs* 174 (86–504) *vs* 535 (257–1213) mg, respectively (*P*<0.001). The median (IQR) steroid exposure (excluding pulsed i.v. methylprednisolone) in the three groups was 37 (26–62) *vs* 158 (82–294) *vs* 375 (244–612) mg, respectively (*P*<0.001). The 90-day mortality was highest in the cohort receiving ECMO 36.6% *vs* 27.3% (IMV) *vs* 8.7% (HFNO/CPAP); *P*=0.002.

No patients receiving HFNO/CPAP support received pulsed dose steroids, whereas 26% of patients supported with IMV and 48.4% of patients supported with ECMO received pulsed dose steroids. Pulsed dose steroids were on average commenced on Day 10 in the IMV group and Day 11 in the ECMO group after ICU admission to our centre.

With regard to the ECMO cohort (Table 2), patients receiving pulsed dose steroids compared with those who did not, were older, with lower baseline CRP, lower PCT, lower lymphocyte count, higher ventilator driving pressure, and worse compliance.

**Table 2:** Comparison of patient characteristics in the ECMO group receiving/not receiving a pulse IVMP. ∗Continuous data are presented as median (IQR). Categorical data presented as counts/total count (%). ^†^Wilcoxon rank sum test; Pearson's χ^2^ test. ^‡^Steroid dose expressed as dexamethasone equivalence in milligrams. APACHE, Acute Physiology and Chronic Health Evaluation; ECMO, extracorporeal membrane oxygenation; IQR, inter-quartile range; IVMP, intravenous methylprednisolone; NA, not available; PBW, predicted body weight; Pulse, receiving >250 mg daily for 3 days.

Characteristic	No IVMP (*n*=48)∗	IVMP (*n*=45)∗	*P*-value^†^
Age (yr)	44 [35, 50]	49 [41, 53]	0.044
Sex – male, *n* (%)	30 [62.5]	33 [73.3]	0.264
Weight kg)	90 [75, 107]	93 [80, 110]	0.503
APACHE II	15 [12, 18.2]	15 [13, 17]	0.985
$Pa_{O_{2}}$/FiO_2_ (kPa)	9.6 [8.0, 12.0]	9.3 [7.9, 10.6]	0.533
C-reactive protein (mg L^−1^)	241 [113, 324]	134 [65, 279]	0.023
Procalcitonin (μg L^−1^)	1.46 [0.30, 4.27]	0.62 [0.20, 1.95]	0.002
Lymphocytes (10×^9^ L^−1^)	0.7 [0.5, 1]	0.5 [0.3, 0.7]	0.005
*Respiratory variables*			
FiO_2_ (%)	0.90 [0.80, 0.96]	0.95 [0.80, 1.00]	0.221
$Pa_{O_{2}}$ (kPa)	8.55 [7.54, 9.48]	8.50 [7.15, 9.60]	0.622
$Pa_{{CO}_{2}}$ (kPa)	7.95 [7.06, 9.12]	7.20 [6.65, 8.87]	0.399
pH	7.31 [7.23, 7.36]	7.35 [7.27, 7.40]	0.149
SaO_2_ (%)	87 [76, 91]	82 [74, 90]	0.241
Lactate (mmol L^−1^)	1.30 [1.00, 1.98]	1.20 [1.00, 1.90]	0.667
Bicarbonate (mmol L^−1^)	26.1 [22.1, 30.3]	27.1 [25.0, 31.0]	0.212
Base excess (mmol L^−1^)	4.6 [1.3, 8.3]	4.2 [1.9, 8.8]	0.440
Ventilatory frequency rate (bpm)	22 [18, 24]	24 [18, 28]	0.220
Tidal volume (ml)	480 [395, 573]	450 [410, 496]	0.263
Tidal volume per PBW (ml kg^−1^)	8.11 [5.92, 9.16	6.88 [6.10, 7.78]	0.150
Peak airway pressure (cm H_2_O)	30 [27, 34]	34 [30, 38]	0.138
PEEP (cm H_2_O)	12 [10, 14]	12 [10, 14]	0.743
Driving pressure (cm H_2_O)	15 [13, 19]	18 [16, 23]	0.041
Minute volume (L min^−1^)	9.25 [8.00, 10.85]	9.80 [7.44, 11.04]	0.803
Compliance (ml cm H_2_O^−1^)	32 [26, 36]	22 [17, 34]	0.025
*Outcome variables*			
ICU length of stay	14 [11, 24]	36 [24, 54]	<0.001
90-day mortality	12 (25.0)	22 (48.9)	0.017
Discharge from hospital after 90 days	35 (72.9)	20 (44.4)	0.005
*Corticosteroid variables*^‡^			
Total steroid exposure	270 [151, 368]	1221 [984, 1394]	<0.001
Steroid duration (days)	13 [7, 21]	30 [20, 48]	<0.001
Average daily steroid dose	19 [13, 25]	39 [26, 49]	<0.001
Total steroids without pulse	270 [151, 368]	604 [382, 765]	<0.001
Total steroid days, no pulse	13 [7, 21]	27 [19, 42]	<0.001
Average daily steroids without pulse	19 [13, 25]	20 [15, 28]	0.255.

In both IMV and ECMO groups (Table 2, Table 3), the length of stay was longer, the 90-day mortality was higher, and the rate of discharge from hospital after 90 days was lower in patients with more severe disease who received pulsed dose steroids.

**Table 3:** Comparison of patient characteristics in the IMV group receiving/not receiving a pulse IVMP. ∗Continuous data are presented as median (IQR). Categorical data presented as counts/total count (%). ^†^Wilcoxon rank sum test; Pearson's χ^2^ test. ^‡^Steroid dose expressed as dexamethasone equivalence in milligrams. APACHE, Acute Physiology and Chronic Health Evaluation; ECMO, extracorporeal membrane oxygenation; IQR, inter-quartile range; IMV, invasive mechanical ventilation; IVMP, intravenous methylprednisolone; NA, not available; PBW, predicted body weight; Pulse, receiving >250 mg daily for 3 days.

Characteristic	No IVMP (*n*=165)∗	IVMP (*n*=58)∗	P-value†
Age (yr)	58 [49, 66]	58 [53, 65]	0.502
Sex – male, *n* (%)	111 [67.2]	42 [72.4]	0.425
Weight (kg)	85 [75, 100]	78 [75, 90]	0.007
APACHE II	14 [12, 18]	15 [12, 17.8]	0.415
$Pa_{O_{2}}$/FiO_2_ (kPa)	16.9 [11.5, 23.0]	12.9 [9.5, 16.8]	<0.001
C-reactive protein (mg L^−1^)	136 [76, 235]	115 [52, 195]	0.138
Procalcitonin (μg L^−1^)	0.59 [0.21, 2.02]	0.63 [0.18, 1.41]	0.386
Lymphocytes (10×^9^ L^−1^)	0.8 [0.5, 1.0]	0.6 [0.4, 0.8]	0.015
*Respiratory variables*			
FiO_2_ (%)	0.70 [0.54, 0.81]	0.70 [0.60, 0.80]	0.844
$Pa_{O_{2}}$ (kPa)	8.64 [7.62,10.22]	8.54 [7.79, 9.44]	0.328
$Pa_{{CO}_{2}}$ (kPa)	6.12 [5.47, 7.31]	5.92 [4.96, 7.46]	0.265
pH	7.36 [7.29, 7.39]	7.37 [7.31, 7.42]	0.094
SaO_2_ (%)	91.3 [89.4, 94.0]	91.3 [88.8, 93.0]	0.364
Lactate (mmol L^−1^)	1.55 [1.20, 2.10]	1.70 [1.33, 2.08]	0.370
Bicarbonate (mmol L^−1^)	25.8 [21.8, 27.9]	24.3 [21.6, 28.8]	0.837
Base excess (mmol L^−1^)	–0.9 [–2.9, 2.9]	–0.5 [–3.2, 3.2]	0.619
Ventilatory frequency (bpm)	20 [18, 24]	21 [18, 27]	0.364
Tidal volume (ml)	548 [486, 632]	502 [448, 623]	0.071
Tidal volume per PBW (ml kg^−1^)	8.63 [7.33, 9.98]	7.96 [7.06, 9.42]	0.084
Maximum PEEP (cm H_2_O)	10 [8, 12]	10 [8, 10]	0.164
Driving pressure (cm H_2_O)	16 [13, 19]	17 [14, 20]	0.414
Minute volume (L min^−1^)	9.20 [7.77,11.00]	8.95 [7.73,10.47]	0.677
Pressure support (cm H_2_O)	12 [8, 15]	12 [8, 18]	0.413
Compliance (ml cm H_2_O^−1^)	33 [24, 48]	27 [21, 45]	0.138
*Outcome variables*			
ICU length of stay	24 [13, 39]	38 [26, 50]	<0.001
90-day mortality	33 (20.0)	28 (48.3)	<0.001
Discharge from hospital after 90 days	130 (78.8)	30 (51.7)	<0.001
*Corticosteroids variables*^‡^			
Total steroid exposure	128 [60, 225]	843 [677, 1024]	<0.001
Steroid duration (days)	15 [8, 21]	34 [24, 45]	<0.001
Average daily steroid dose	9 [7, 13]	27 [20, 35]	<0.001
Total steroids without pulse	128 [60, 225]	351 [213, 473]	<0.001
Total steroid days without pulse	15 [8, 21]	30 [20, 41]	<0.001
Average daily steroid dose without pulse	9 [7, 13]	12 [10, 15]	<0.001

### Association between pulsed corticosteroids and outcome

Variables associated with the odds of receiving pulsed dose steroid (Table 4) were evaluated in a univariable logistic regression model.

**Table 4:** IVMP pulse univariable logistic regression model. Model constructed with administration of IVMP pulse (yes/no) the dependent variable. Appropriate covariates were selected and univariable logistic regression models constructed for each covariate. *n*, number of patients with available data. 95% CI, 95% confidence interval; IVMP, intravenous methylprednisolone; PBW, predicted body weight.

Characteristic	*n*	Odds ratio	95% CI	*P*-value
$Pa_{O_{2}}$/FiO_2_ (kPa)	343	0.91	0.87, 0.94	<0.001
Procalcitonin (μg L^−1^)	351	0.95	0.89, 0.99	0.048
Lymphocyte count (10×^9^ L^−1^)	362	0.38	0.20, 0.69	0.003
FiO_2_ (%)	341	5.43	1.47, 20.9	0.012
$Pa_{{CO}_{2}}$ (kPa)	342	1.13	1.00, 1.28	0.045
SaO_2_ (%)	321	0.96	0.93, 0.99	0.008
Bicarbonate (mmol L^−1^)	339	1.05	1.00, 1.09	0.044
Base excess (mmol L^−1^)	340	1.07	1.03, 1.13	0.002
Tidal volume (ml)	299	1.00	1.00, 1.00	0.013
Tidal volume per PBW (ml kg^−1^)	289	0.82	0.72, 0.93	0.003
Driving pressure (cm H_2_O)	294	1.05	1.01, 1.10	0.025

Significant associations were demonstrated for $Pa_{O_{2}}$/FiO_2_ ratio, PCT, lymphocytes, FiO_2_, $Pa_{{CO}_{2}}$, SaO_2_, bicarbonate concentration, base excess, tidal volume, tidal volume/body weight, and driving pressure. A second univariable logistic regression model was performed for 90-day mortality (Table 5). Statistically significant associations were shown between 90-day mortality and the use of pulsed dose steroids (odds ratio [OR]=4.0; 95% confidence interval [CI], 2.5–6.7; *P*<0.001). When corrected for age, APACHE II score, $Pa_{O_{2}}$/FiO_2_ ratio, and total exposure to steroids without a pulsed dose of methylprednisolone (Table 6), pulsed steroid was still associated with higher mortality (OR=3.0; 95% CI, 1.5–6.3; *P*=0.003).

**Table 5:** Ninety-day mortality univariable logistic regression model. Model constructed with 90-day mortality as the dependent variable. Appropriate covariates were selected and univariable logistic regression models constructed for each covariate. 95% CI, 95% confidence interval; APACHE, Acute Physiology and Chronic Health Evaluation; IVMP, intravenous methylprednisolone; *n*, number of patients with available data.

Characteristic	*n*	Odds ratio	95% CI	*P*-value
IVMP pulse	362	4.04	2.47, 6.67	<0.001
Age (yr)	362	1.04	1.02, 1.06	<0.001
Sex – male, *n* (%)	362	2.50	1.44, 4.53	0.002
APACHE II	362	1.15	1.10, 1.22	<0.001
$Pa_{O_{2}}$/FiO_2_ ratio (kPa)	343	0.88	0.83, 0.92	<0.001
FiO_2_ (%)	341	4.08	1.09, 15.9	0.039
$Pa_{O_{2}}$ (kPa)	342	0.82	0.70, 0.95	0.011
pH	343	0.04	0.00, 0.40	0.007
Lactate (mmol L^−1^)	340	1.30	1.02, 1.68	0.036
PEEP (cm H_2_O)	280	1.12	1.03, 1.23	0.014

**Table 6:** Ninety-day mortality multivariable logistic regression model. Model constructed with 90-day mortality as the dependent variable. Appropriate covariates were selected and multivariable logistic regression models constructed for each covariate. 95% CI, 95% confidence interval; APACHE, Acute Physiology and Chronic Health Evaluation; IVMP, intravenous methylprednisolone.

Characteristic	Odds ratio	95% CI	*P*-value
Age (yr)	1.06	1.03, 1.09	<0.001
APACHE II	1.11	1.03, 1.19	0.004
$Pa_{O_{2}}$/FiO_2_ ratio (kPa)	0.88	0.82, 0.92	<0.001
Total steroids dose without pulse	2.76	0.68, 11.4	0.156
IVMP pulse	3.03	1.48, 6.28	0.003

## Discussion

This study reports patient characteristics, corticosteroid usage, and outcomes in a cohort of patients with COVID-19 receiving a spectrum of respiratory support ranging from HFNO/CPAP to ECMO.

The main findings are: (1) the dose of steroids administered increased with the disease severity and 90-day mortality was highest in the cohort receiving ECMO; (2) administration of ‘pulsed’ steroids was frequent in patients with more severe disease; (3) pulsed steroids were associated with higher 90-day mortality when corrected for age and severity of disease.

### Steroids, inflammation, and disease severity

In our patient population, the levels of CRP on admission to hospital in patients with COVID-19 were closely related to the severity of illness. Tan and colleagues^18^ demonstrated that patients with more severe COVID-19 had higher initial CRP, and levels of CRP predicted the likelihood of early severe COVID-19. In our study, the median CRP increased with the severity of organ failure and need for more advanced organ supportive therapies.^18^^,^^19^ The median total dexamethasone-equivalent steroid exposure for patients in the HFNO/CPAP group was lower than reported in the RECOVERY (60 mg) and COVID STEROID 2 studies (60 *vs* 120 mg),^4^^,^^20^ whereas doses reported in the IMV group were comparable with those in the DEXA-ARDS and CoDex studies (150 mg).^5^^,^^9^ Patients in the ECMO group received considerably more steroids compared with other studies,^4^^,^^5^^,^^9^^,^^20^ but less than in the protocol proposed by Meduri and colleagues^8^ for moderate-to-severe ARDS. Daily doses described in the study are consistent with those described in the literature for COVID-19 and non-COVID-19 ARDS.^4^, ^5^, ^6^^,^^8^^,^^9^

Although the RECOVERY trial used dexamethasone 6 mg for 7–10 days in patients with COVID-19 requiring supplementary oxygen,^21^, ^22^, ^23^ the optimal dose of steroids remains controversial, with some studies suggesting that a higher dose might be beneficial in higher severity disease.^5^^,^^9^ One study that demonstrated no benefit from higher dose, the COVID STEROID 2 trial compared dexamethasone 12 *vs* 6 mg in patients with COVID-19 requiring at least 10 L min^−1^ of oxygen or mechanical ventilation.^20^ Although no difference was found in mortality at 28 days, or median number of days alive without life support, a pre-planned Bayesian analysis found a higher probability of benefit and a lower probability of harm for the 12 mg group.^24^

In our study, the median dexamethasone-equivalent dose and duration of treatment for HFNO/CPAP, IMV, and ECMO groups were 7 mg for 6 days, 11 mg for 18 days, and 25 mg daily for 20 days, respectively, which is comparable with other published studies.^4^^,^^5^^,^^9^^,^^20^ Conflicting results have been shown by other studies. In a recent meta-analysis including 12 studies comparing high-dose *vs* low-dose steroids in COVID-19 patients, no significant difference in mortality was found between the high- and low-dose corticosteroids groups.^25^ However, high dose corticosteroids were dichotomously defined as >20 mg dexamethasone daily and a wide range of disease severity was included.^25^

The impact of timing and duration of corticosteroid therapy in COVID-19 has not been well defined. In the landmark COVID-19 trials of steroids the duration was relatively short (up to 10 days in RECOVERY, 7 days in COVID STEROID 2) and there was no dose tapering.^4^^,^^20^ However, in non-COVID ARDS studies, there is a suggestion that corticosteroid administration should be relatively prolonged (up to 28 days) with a tapering period.^9^^,^^26^^,^^27^ Abrupt tapering of corticosteroids has been associated with late deterioration and poorer outcome in ARDS, which blunted the apparent efficacy of steroids in a re-analysis of the Late Steroid Rescue Study (LaSRS) trial of methylprednisolone for late (>7 days) ARDS.^27^ Associations have been made between a greater likelihood of deterioration after cessation of steroids in COVID-19 with initial disease severity or higher inflammatory markers at the point of cessation.^28^ Consequently, prolonged courses of steroid for patients with severe disease, as clinicians utilised in this cohort, may be rational, but has not yet been prospectively evaluated. Tapering of steroids was standard practice in our institution, particularly for higher doses over a prolonged period of time. In our study, the median duration of treatment in the HFNO/CPAP group was less (7 days) compared with other published studies but longer in the IMV (18 days) and ECMO (20 days) groups.^4^^,^^9^^,^^20^

### Pulsed steroid administration

In our institution, the decision to prescribe pulsed i.v. methylprednisolone was made by a multi-disciplinary team including respiratory physicians, rheumatologist with special interest in interstitial lung disease, infection disease specialists, radiologists, and intensivists, after a review of CT imaging (e.g. pattern compatible with organising pneumonia or disease evolution), ventilation, and biochemical variables. Patients received pulsed methylprednisolone if they showed signs of persisting inflammation, had no evidence of active infection at the time of the initiation of methylprednisolone, and had not improved with the lower dose steroid regimen. In this setting, the use of methylprednisolone was essentially a salvage approach for patients who remained critically ill with progressive C-ARDS. In our study, pulsed methylprednisolone doses were 0.5–1 g daily (weight-adjusted) for 3 days followed by dexamethasone 20 mg daily for 5–7 days. In our cohort, almost half of patients on ECMO and one-quarter of mechanically ventilated patients received pulsed dose steroids. We found strong associations between markers of disease severity and the likelihood of receiving a pulsed dose steroids, and this group of patients had higher 90-day mortality. However, it is unclear whether pulsed steroids *per se* increased the risk of death or whether the patients selected to receive pulsed steroids had the highest risk of death because of baseline severity or failure to respond to supportive treatment and standard pharmacological treatment and initial steroid dose.

The evidence of benefit for pulsed i.v. methylprednisolone in either ARDS or C-ARDS context is limited. Edalatifard and colleagues^14^ demonstrated that i.v. methylprednisolone 250 mg daily for 3 days plus standard care *vs* standard care alone at the beginning of the early pulmonary phase of illness improved oxygen saturation, symptoms of dyspnoea, and inflammatory markers, and reduced the mortality rate. However, standard care did not include other corticosteroids, patients did not undergo tracheal intubation, the study was a single-blind design, and there was a limited sample size.^14^ In our study, we included patients receiving mechanical ventilation and noninvasive ventilation, and our standard care was dexamethasone in all groups. Salvarani and colleagues^29^ recently published the first randomised trial comparing i.v. methylprednisolone 1 g daily for 3 days or placebo in addition to standard dexamethasone (6 mg daily for 10 days) in COVID-19. They enrolled patients with CRP >10 times the upper reference limit of normal but >80% of patients never required admission to critical care and most were managed with supplementary oxygen at baseline. Importantly, patients receiving mechanical ventilation were not enrolled. There was no significant benefit of pulsed methylprednisolone with regard to progression of respiratory support, recovery, or mortality.^29^ However, because of the low sample size, modest but clinically significant effects cannot be excluded, and arguably such intensified doses of corticosteroids should only be considered in patients with severe and deteriorating disease despite RECOVERY dose corticosteroids. Monreal and colleagues^30^ published a single-centre study comparing the short-term use of high-dose methylprednisolone (≥250 mg day^−1^) or standard dose ≤1.5 mg kg^−1^ day^−1^ in severe COVID-19 with ARDS. They reported higher mortality and higher risk of need for mechanical ventilation in the high dose group, particularly in older patients, but the high-dose group had worse baseline respiratory function, were older and with more comorbidities.^30^

### Limitations and strengths

Our study has several limitations: (1) this was a single-centre retrospective analysis with no standardised protocol for the use of pulsed steroids; (2) data on corticosteroids received before admission or after discharge from our ICU was not available for analysis; (3) markers of severity including mechanical ventilation and inflammatory markers analysed refer to Day 1 of ICU admission and may not be reflective of severity at the time of decision-making for pulsed methylprednisolone; (4) the risk of selection and treatment bias cannot be ascertained and adjusted and therefore the causality of steroid use on mortality needs prospective evaluation as patients who received pulsed methylprednisolone may represent a subset with a different disease phenotype. However, it is also important to note that patients who did not improve with initial doses of steroids and received methylprednisolone did have survival of more than 50% in the IMV and ECMO groups.

The strengths of this study are that it investigates a large number of patients receiving a wide spectrum of C-ARDS disease severity; it also provides a comparison of organ support ranging from HFNO/CPAP to ECMO which is not otherwise available in the existing literature. The reporting of cumulative dose of corticosteroids is another strength made possible by an electronic patient system that allows recording of detailed data on medication doses and timing of administration.

## Conclusions

Despite strong evidence in support of corticosteroids for patients with COVID-19 pneumonia, the optimal dose of corticosteroids is still unclear. Future randomised trials comparing different corticosteroid regimens according to the degree of inflammation and severity of disease are necessary.

Corticosteroid exposure increased with C-ARDS severity and 90-day mortality was highest in the cohort receiving ECMO. Pulsed dose steroid use was more frequent in patients receiving high respiratory support. Future studies should address patient selection and outcome effects of pulsed steroids in severe and deteriorating patients with COVID-19 pneumonia.

## Authors’ contributions

Study design and analysis: CR, FH, LC.

Patient recruitment, data collection, and writing up of the first draft of the paper: CR, LC.

All other authors were involved in data interpretation, writing of the manuscript, and approval of the final version of the manuscript.

## Declaration of interest

The authors declare they have no conflicts of interest.

## Funding

CR has received research fellowship funding from National Institute for Health and Care Research to assist with the completion of this study analysis and write-up of the manuscript (reference: NIHR302687).
